# Parental Acceptance and Preferences Regarding Advanced Versus Basic Behavior Management Techniques in Pediatric Dentistry: A Systematic Review and Meta-Analysis of Observational Studies from the Middle East

**DOI:** 10.3390/healthcare14172729

**Published:** 2026-08-26

**Authors:** Faisal Abed, Khlood Baghlaf, Shahad N. Abudawood, Heba Jafar Sabbagh

**Affiliations:** 1 Specialized Dental Center, Ministry of Health, Madinah 42315, Saudi Arabia; 2 Pediatric Dentistry Department, Faculty of Dentistry, King Abdulaziz University, Jeddah 21589, Saudi Arabia; kbaghlaf@kau.edu.sa (K.B.); hsabbagh@kau.edu.sa (H.J.S.)

**Keywords:** parental acceptance, parental preferences, behavior guidance techniques, Tell-Show-Do, nitrous oxide, general anesthesia, protective stabilization, pediatric dentistry, Middle East, observational studies

## Abstract

**Objective:** The aim of this systematic review was to evaluate evidence comparing parental acceptance and preferences of advanced behavior management techniques with basic behavior management techniques among pediatric dental populations in the Middle East. **Methodology:** A systematic review and meta-analysis of observational studies was conducted in accordance with PRISMA 2020 guidelines and registered with PROSPERO (CRD420261294758) in February 2026. The systematic review included studies published between January 2015 and February 2026, identified through PubMed, Cochrane Library, Web of Science, ScienceDirect, and Google Scholar. Inclusion criteria encompassed observational studies assessing parental acceptance of behavior guidance techniques for healthy children aged 2–14 years, conducted in the Middle East. Two authors independently selected the studies and extracted the data. The risk of bias was assessed independently by two reviewers (F.A. and K.B) using an adapted Newcastle–Ottawa Scale (NOS), with any uncertainties resolved through discusscoion with a third reviewer (H.J.S.). Comparing the different behavior-guided techniques allowed for the creation of pooled effect estimates for the included studies. **Result:** A total of 88 studies were identified from the database search. Of the 88 studies identified, 11 met the inclusion criteria. Eleven cross-sectional observational studies were conducted in Iran, Turkey, Saudi Arabia, Bahrain, Egypt, Jordan, and Kuwait. The TSD approach consistently garnered the highest parental acceptance, ranging from 88% to 98%. In contrast, advanced methods like GA and physical restraint were the least preferred. Six meta-analyses were conducted across four clinical comparisons. Parental acceptance of GA was significantly lower than TSD (OR = 0.03, 95% CI: 0.02–0.07, *p* < 0.00001), as was parental preference (OR = 0.08, 95% CI: 0.05–0.15, *p* < 0.00001), with an overall pooled OR of 0.04 (95% CI: 0.02–0.08) and a complementary continuous analysis (SMD = −1.03, 95% CI: −1.54 to −0.51). Similarly, parental acceptance of protective stabilization was significantly lower than TSD (OR = 0.10, 95% CI: 0.06–0.15, *p* < 0.00001), as was parental preference (OR = 0.05, 95% CI: 0.03–0.08, *p* < 0.00001), with an overall pooled OR of 0.07 (95% CI: 0.05–0.11) and SMD of −1.87 (95% CI: −2.32 to −1.43). Comparisons involving N_2_O demonstrated extreme heterogeneity (I^2^ = 95%), precluding reliable pooled estimates. Acceptance of N_2_O varied more, influenced by regional differences and contextual factors. **Conclusions:** Middle Eastern populations of parents were consistently less accepting of advanced behavior management techniques, especially GA and protective stabilization, compared with TSD. These results are considered preliminary and are mainly based on cross-sectional studies and should be interpreted in the context of moderate risk of bias and substantial heterogeneity across the included studies. There is a need for more high-quality, longitudinal, multicenter studies to improve the evidence base for culturally sensitive behavior guidance in pediatric dental practice.

## 1. Introduction

The efficacy of pediatric dental treatment primarily relies on the behavior-guiding technique (BGT) employed [1]. BGT alleviates anxiety and fear, promotes a positive attitude, and provides oral health care with physical and mental confidence for children, irrespective of special health care needs (SHCN) [1]. The dentist’s methodology must be incorporated into the comprehensive application of BGT, considering the uniqueness of children, the practitioners’ competencies, and the parents’ perspectives [2]. Considering the societal transformations in recent years, an increasing number of parents and siblings accompany children to their dental screenings [3], reflecting a pronounced desire among families to engage in treatment decision-making. The perspectives of contemporary parents have impacted the utilization of BGT [4]. The methods used by the dental team have changed throughout time to reflect changes in society and families [4].

The American Academy of Pediatric Dentistry (AAPD) currently classifies BGTs into two broad categories: basic and advanced. Basic behavior guidance encompasses communication and communicative guidance, positive pre-visit imagery, direct observation, Tell-Show-Do (TSD), Ask-Tell-Ask, voice control, nonverbal communication, positive reinforcement and descriptive praise, distraction, memory restructuring, parental presence/absence, communication techniques for parents and age-appropriate patients, and nitrous oxide/oxygen inhalation (N_2_O). Each basic behavior management technique serves a specific purpose and should be selected according to the patient’s individual needs, particularly for children with limited emotional or psychological maturity or with mental, physical, or medical conditions [5]. Parental acceptance is defined as parents’ willingness to consent or approve the use of a specific behavior management technique for their child’s dental treatment [1]. Parental preference, by contrast, refers to the relative prioritization or ranking of one technique over another, reflecting the parents’ values, cultural background, and beliefs regarding their child’s dental care [6]. Both constructs are important clinical considerations, as parental attitudes toward behavior management techniques directly influence treatment planning, informed consent, and the therapeutic relationship between the dental team and the family [1]. Advanced behavior guidance techniques include protective stabilization, which may be active, involving physical assistance by another person; passive, involving the use of a stabilization device; or a combination of both [1], as well as pharmacological approaches, such as procedural sedation and general anesthesia (GA) [5]. Given their complexity and potential risks, these techniques should be used cautiously and require appropriate specialized training, typically obtained through a postgraduate pediatric dentistry residency program. Furthermore, pharmacological approaches may be indicated to protect the child’s developing psyche and overall welfare, to facilitate the safe delivery of definitive and durable dental treatment, or to address medical conditions that necessitate such an approach [7].

Procedural sedation is a medication-induced state that may range from minimal sedation, or anxiolysis, to moderate or deep sedation. When appropriately administered, it can be a safe and effective approach for children who are unable to cooperate with dental treatment [8]. When appropriately used, sedation or GA for comprehensive dental rehabilitation may improve children’s oral health-related quality of life [7].

Certain patients experience significant challenges in cooperating throughout dental visits, rendering the exclusive use of non-pharmacological treatments inadequate [1]. More intrusive procedures might create clinical scenarios of heightened stress, necessitating enhanced performance from the professional in controlling a child’s behavior. Such instances may require more stringent methodologies [1]. In these instances, behavioral counseling can be tailored to the patient’s requirements and the parents’ preferences [5]. Given that parents’ perspectives on BGT utilization influence the treatment plan, it is essential to examine their ideas while determining BGT application priority [6].

Furthermore, the AAPD recognizes that cultural factors and differences in parenting styles across families and communities may influence both children’s behavior during dental visits and the selection or acceptance of BGTs [7,8,9]. Therefore, the aim of this systematic review was to evaluate evidence comparing parental acceptance of and preferences for advanced behavior management techniques with basic behavior management techniques among pediatric dental populations in the Middle East.

### PECO

The studies included in this review were selected by the PECO elements: Population (P): Parents (mothers or fathers) of healthy children (ASA I) in the Middle Eastern population. Exposure (E): Advanced behavior management techniques in pediatric dentistry (conscious sedation, general anesthesia, active protective stabilization, passive protective stabilization, physical restraint, and papoose-board restraint). Comparison (C): Two basic behavior management techniques, “Tell-Show-Do or nitrous oxide”. Outcome (O): Parental acceptance or preference for behavior management techniques.

The research question investigated is as follows: “How do parents of healthy children aged 2 to 14 in the Middle East feel about the use of advanced behavior management techniques like sedation, general anesthesia, and protective stabilization compared to basic behavior management techniques such as Tell-Show-Do and nitrous oxide during pediatric dental treatment?”

## 2. Materials and Methods

### 2.1. Search Strategy

A systematic review and meta-analysis of observational studies was performed based on the Preferred Reporting Items for Systematic Reviews and Meta-Analyses (PRISMA) 2020 guidelines [10] (Supplementary Materials). The review was prospectively registered with the International Prospective Register of Systematic Reviews (PROSPERO) database (CRD420261294758) on 22 February 2026 prior to data extraction and meta-analysis.

A search was conducted in February 2026 for relevant studies published between 1 January 2015 and February 2026. We searched the following electronic databases without language restrictions: PubMed, Cochrane Library, Web of Science, ScienceDirect, and Google Scholar.

The search strategy was adapted to the syntax requirements of each database as follows.

In PubMed, the following search string was applied using a combination of MeSH terms and free-text keywords with Boolean operators: ((parents[MeSH] OR mother[tiab] OR father[tiab] OR caregiver[tiab]) AND (oral sedation[tiab] OR sedation[MeSH] OR “general anesthesia”[MeSH] OR papoose[tiab] OR “active restraint”[tiab] OR “passive restraint”[tiab] OR “physical restraint”[MeSH] OR “protective stabilization”[tiab]) AND (“tell-show-do”[tiab] OR “nitrous oxide”[MeSH]) AND (acceptance[tiab] OR preference[tiab] OR attitude[tiab])). MeSH terms applied included the following: parents, conscious sedation, anesthesia general, restraint physical, nitrous oxide, and patient acceptance of health care.

In Web of Science, the basic search terms were applied to titles, abstracts, and keywords using the topic search field (TS=): TS = ((parents OR mother OR father) AND (oral sedation OR general anesthesia OR papoose OR active restraint OR passive restraint OR physical restraint OR protective stabilization) AND (Tell-Show-Do OR nitrous oxide) AND (acceptance OR preference)).

In ScienceDirect, the advanced search option was used with the following terms: (parental acceptance OR parental preference) AND (behavior management OR behaviour management) AND (pediatric dentistry OR paediatric dentistry) AND (Tell-Show-Do OR nitrous oxide OR general anesthesia OR protective stabilization), filtered by article type (research articles and review articles) and publication year (2015–2026).

In the Cochrane Library, the following search string was applied: (parent OR mother OR father) AND (sedation OR “general anesthesia” OR “protective stabilization” OR “nitrous oxide” OR “papoose board” OR “physical restraint”) AND (“tell-show-do” OR “behavior management” OR “behaviour management”) AND (acceptance OR preference), limited to January 2015 to February 2026. No additional studies meeting the inclusion criteria were identified from the Cochrane Library beyond those retrieved from the other databases.

In Google Scholar, the following keyword string was applied, adapted to the platform’s limitations, which do not support MeSH terms or advanced Boolean syntax: (“parental acceptance” OR “parental preference”) AND (“behavior management” OR “behaviour management”) AND (“pediatric dentistry” OR “paediatric dentistry”) AND (“tell-show-do” OR “nitrous oxide” OR “general anesthesia” OR “protective stabilization”); the first 200 results were screened by title and abstract and sorted by relevance, with the date range restricted to January 2015 to February 2026 using the platform’s built-in filter.

The inclusion criteria were as follows: observational studies including case-control, cohort, and cross-sectional studies with comparison groups; studies assessing parental acceptance of advanced behavior management techniques in pediatric dentistry; and studies involving healthy children based on the American Society of Anesthesiologists (ASA I) Physical Status [11] aged 2 to 14 years across the Middle East. For the purposes of this review, the Middle East was defined consistent with established academic classifications used in previously published systematic reviews in the region [12] encompassing Saudi Arabia, Kuwait, Bahrain, Jordan, Egypt, Iran, and Turkey. Exclusion criteria included studies without comparison groups (e.g., case reports, expert opinions, conference abstracts), studies not involving pediatric patients, studies focusing on dentists’ perspectives rather than parental acceptance, and studies evaluating basic behavior techniques without comparison to an advanced technique.

### 2.2. Study Selection and Data Extraction

Two reviewers (F.A. and K.B.) independently assessed the titles and abstracts of all identified studies to determine if they met the inclusion criteria. The full-text articles of the selected studies were further evaluated by the same reviewers. In studies with the same sample, those with more data were preferred. Study design, country, sample size, children’s ages, assessment methods, assessment tools, and outcomes were extracted. Additional published papers were identified by checking cited references in the included abstracts. Finally, the full-text articles were screened against the pre-specified inclusion and exclusion criteria, and if any uncertainty existed about an article, a third reviewer was assigned.

### 2.3. Assessment of the Risk of Bias

Two independent reviewers (F.A. and K.B.) assessed the methodological quality of the included studies using an adapted version of the Newcastle-Ottawa Scale (NOS) for cross-sectional studies [13,14]. Any disagreements were resolved through discussion with a third reviewer (H.J.S.). The adapted (NOS) maintained the three core domains: selection, comparability, and outcome, with adaptations for non-applicable items given the cross-sectional and vignette-based nature of the included studies. The tool scores from 0 to 10 and evaluates studies in three domains: the adequacy of case and control selection and their representativeness of the wider population; comparability of cases and controls based on study design or analysis methods; and accuracy of exposure assessment. Total scores were used to classify studies as low risk of bias (>6), moderate risk (4–6), or high risk (≤3). It gave a systematic and transparent evaluation of study quality. We recognize that the NOS as adapted may not completely reflect the methodological nuances of vignette- and video-based parental preference studies and that the certainty of the adapted scoring may be less robust than the original NOS designed for cohort and case-control studies [13,14].

### 2.4. Meta-Analysis

Meta-analyses were conducted when at least two eligible studies were available for a given comparison, using RevMan Web (10.1.0). A two-sided significance level of 0.05 was applied throughout. Pooled effect estimates were expressed as odds ratios (ORs) with 95% confidence intervals (CIs) and calculated using the Mantel–Haenszel method.

For studies that directly reported binary outcomes, the number of parents who accepted each behavior management technique was defined as the number of events, while the remaining participants were classified as non-events. These data were entered into RevMan Web as dichotomous outcomes.

To harmonize outcomes reported using visual analog scales (VASs) or questionnaire scales, and not mentioning the percentage of parental acceptance scores, these scores were converted into binary acceptance outcomes when sufficient data were available. Scores were expressed as a percentage of the maximum possible score. Parents who assigned a score of 75% or higher to a behavior management technique were classified as accepting the technique, whereas those who assigned a score below 75% were classified as not accepting it.

When the published data were insufficient to determine the number of participants who scored 75%, the study was excluded from the binary quantitative synthesis. For studies that directly reported acceptance frequencies or percentages, the reported values were used without further transformation.

The choice between fixed-effect and random-effects models was based on the degree of statistical heterogeneity, assessed using the Chi-square test and quantified using the I^2^ statistic. A fixed-effect model was applied when heterogeneity was low and non-significant, defined as *p* > 0.10 and I^2^ < 50%. A random-effects model was applied when substantial heterogeneity was identified, defined as *p* ≤ 0.10 and/or I^2^ ≥ 50%. In borderline cases, a random-effects model was used as a more conservative approach [15].

To explore potential sources of heterogeneity, pre-specified subgroup analyses were conducted where applicable. For binary outcome analyses, studies were grouped by outcome measure type: parental acceptance studies reporting number or percentage of parents accepting each technique versus parental preference studies reporting ranked or preferred technique choices. For continuous outcome analyses, studies were grouped by data reporting format: studies reporting total sample means versus studies reporting sex-stratified means. Subgroup differences were assessed using the Chi^2^ test, with *p* < 0.05 indicating statistical significance.

## 3. Results

**Figure 1:**
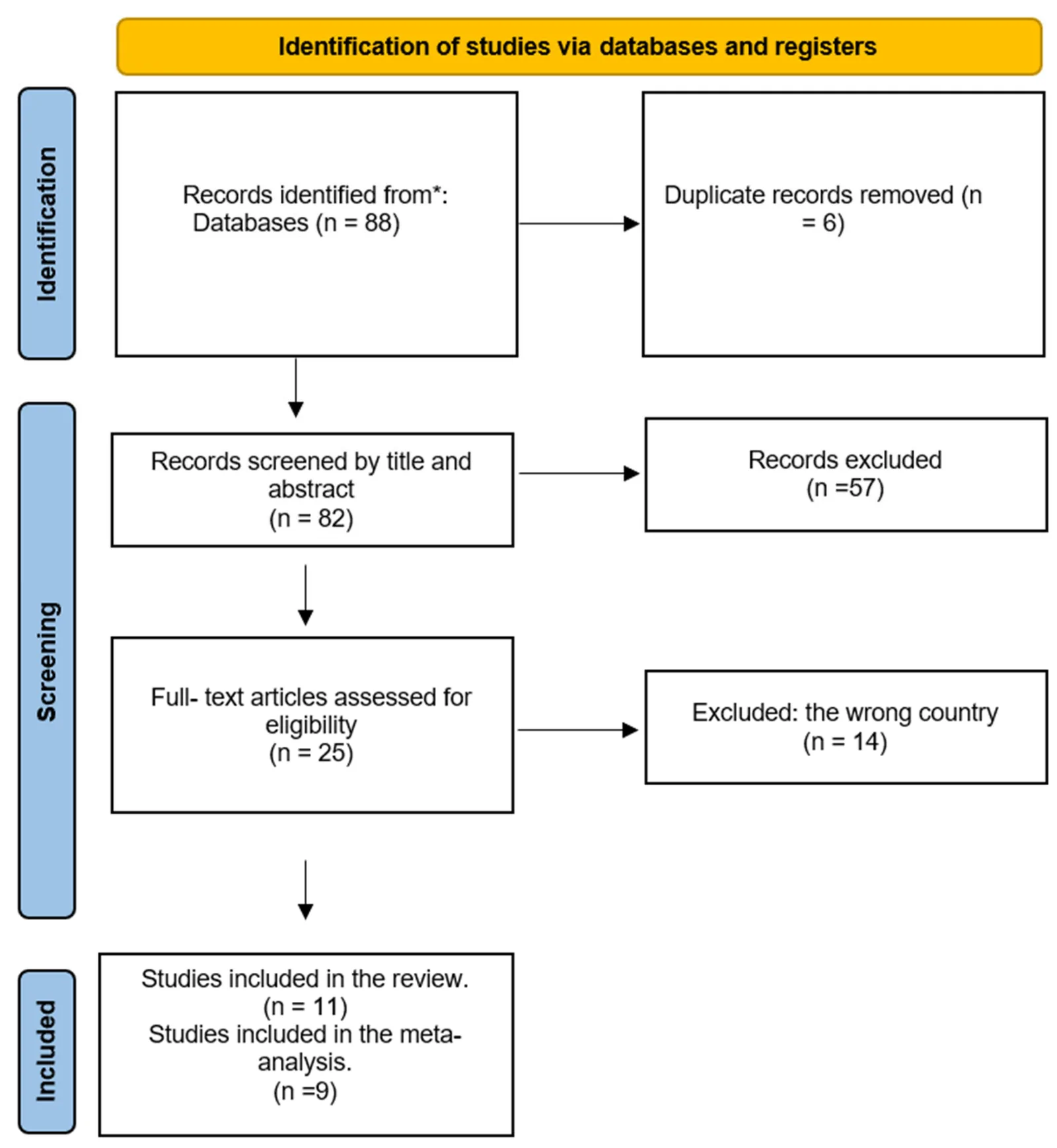
PRISMA 2020 flow diagram. Adapted from Page et al. [10]. * Records were identified from the following electronic databases: PubMed (n = 20), Google Scholar (n = 35), Web of Science (n = 12), ScienceDirect (n = 10), Cochrane Library (n = 11).

The database search resulted in 88 hits. All database search results were exported to EndNote (Clarivate Analytics, Philadelphia, PA, USA), where duplicate records were identified and removed. The deduplicated records were then imported for title and abstract screening. The deduplicated records were then screened based on their titles and abstracts. After eliminating duplicates, 82 remained. A review of titles and abstracts identified 25 full-text articles as potentially eligible for comparison. Of these, 14 full-text articles were excluded because they were not conducted in the Middle East as defined by the inclusion criteria, and one did not compare the advanced BMG. Therefore, there were 11 articles that fulfilled the inclusion and exclusion criteria and qualified for this systematic review (Figure 1) [16,17,18,19,20,21,22,23,24,25].

The review comprised eleven cross-sectional observational studies assessing parental acceptance of advanced behavior guidance techniques, including GA, sedation, or physical or protective restraint. These advanced behavior guidance techniques were compared to TSD and N_2_O in eleven studies. The sample comprised 1409 parents from several countries, including Iran, Turkey, Saudi Arabia, Bahrain, Egypt, Jordan, and Kuwait. Most of the studies used videos or pictures to show the BGTs to parents, who then used visual analog scales (VASs) or structured questionnaires to rate how much they liked them. The acceptance of advanced behavior techniques ranged from 9% [23] to 51% [21] for GA and from 16.9% to 100% [24] for active or passive restraint. There were eight studies that compared advanced behavior techniques with TSD [16,19,20,21,22,23,25], and five that compared them with N_2_O [16,17,18,24,26]. The characteristics of the included studies are presented in Table 1 and Table 2.

Parental acceptance or preference of advanced behavior guidance techniques compared with Tell–Show–Do (TSD) is presented in Table 2. All studies assessed parental acceptance or preference among parents with no prior experience of the evaluated techniques.

Three studies were conducted in Iran. Jafarzadeh et al. (2015) included 54 parents [21], of whom 57% accepted active restraint, 54% accepted oral sedation, 51% accepted general anesthesia (GA), and 35% accepted passive restraint, compared with 94% who accepted TSD. The differences were statistically significant (*p* < 0.001). Similarly, Jahanimoghadam et al. reported lower mean parental acceptance scores for GA (mean = 65, SE = 3.9) and physical restraint (mean = 50, SE = 4.3) than for TSD (mean = 80, SE = 2.3). These differences were statistically significant (*p* = 0.001 and *p* < 0.001, respectively) [22]. In addition, Hashemi et al. (2021) reported significantly lower mean parental acceptance scores for GA (4.52 ± 2.17), sedation (3.92 ± 1.13), and physical restraint (2.47 ± 1.34) than for TSD (6.30 ± 2.47; *p* < 0.001) [20].

Two studies were conducted in Turkey. Taran et al. (2018) [25] included 142 parents, of whom 25.4% preferred GA and 16.9% preferred protective stabilization. Both techniques were significantly less preferred than TSD, which was preferred by 80.3% of parents (*p* < 0.001) [25]. Similarly, Candan et al. (2023) reported parental preferred rates of 50.0% for N_2_O, 41.9% for protective stabilization, and 30.1% for GA, compared with 94.1% for TSD [19].

In Saudi Arabia, among 130 participating parents, 56.2% accepted protective stabilization and 47.7% accepted GA, compared with 93.1% who accepted TSD. The differences were statistically significant (*p* < 0.001) [26]. In a Bahraini cross-sectional study, TSD was accepted by 98% of parents, compared with 71% for physical restraint and 67% for GA (*p* < 0.001) [16]. Finally, in a study conducted in Cairo, Egypt, 88% of parents accepted TSD, compared with 47% who accepted physical restraint and only 9% who accepted GA [23].

Parental acceptance of advanced behavior guidance techniques compared with nitrous oxide (N_2_O) is presented in Table 3. All included studies used cross-sectional designs with a comparison group. Furthermore, all studies assessed parental acceptance among parents with no prior experience of the evaluated techniques, except Sabbagh et al. (2021) [24], which was conducted in Saudi Arabia. In that study, the children of the participating parents had received the behavior guidance techniques being evaluated.

Sabbagh et al. (2021) [24] reported 100% parental acceptance of the papoose board, compared with 96.2% acceptance of N_2_O, with no statistically significant difference between the two techniques (*p* = 1.00). However, the combined use of the papoose board and N_2_O resulted in a significantly lower acceptance rate (81.2%) compared to using N_2_O alone (*p* = 0.04).

In Kuwait, Alkandari et al. (2016) [18] conducted a cross-sectional study involving 376 parents who completed a self-administered questionnaire. Of these parents, 36% preferred general anaesthesia (GA) for dental treatment, compared with 64% who preferred N_2_O (*p* < 0.001).

In Jordan, Al Zoubi et al. (2021) [17] conducted a photo-based cross-sectional study that measured parental acceptance using Likert-scale scores. Parents rated GA significantly lower than N_2_O (2.11 ± 1.30 vs. 3.22 ± 1.50, *p* < 0.001). Parental acceptance of passive restraint was also significantly lower than that of N_2_O (2.52 ± 1.50 vs. 3.22 ± 1.50, *p* = 0.001). However, the difference in parental acceptance between active restraint and N_2_O was not statistically significant (3.08 ± 1.33 vs. 3.22 ± 1.50, *p* = 0.40).

Three studies used video-based presentations to assess parental acceptance: one conducted in Saudi Arabia involving 130 parents [26], one in Egypt involving 140 parents [23], and one in Bahrain involving 140 parents [16]. In the Saudi Arabian study, parental acceptance rates for physical restraint and GA were 56.2% and 47.7%, respectively. Both rates were significantly lower than the acceptance rate for N_2_O, which was 75.4% (*p* = 0.003 and *p* < 0.001, respectively) [26].

Similarly, in the Egyptian study, parental acceptance of physical restraint was 47%, whereas acceptance of GA was only 9%. Both rates were lower than the parental acceptance rate for N_2_O, which was 88%, with statistically significant differences reported (*p* < 0.001) [23].

In contrast, the Bahraini study reported higher parental acceptance rates for protective stabilization (71%) and GA (76%) than for N_2_O (54%). The differences between N_2_O and protective stabilization and between N_2_O and GA were statistically significant (*p* = 0.003 and *p* = 0.02, respectively) [16].

### 3.1. Study Quality and Risk of Bias

Based on the adapted NOS scoring, three studies were considered to have a low risk of bias (scores >6) (Table 3). The remaining eight studies were judged to have moderate risk of bias (scores 4–6) (Table 3). No studies were classified as high risk (score ≤ 3).

The most common source of bias was in the selection domain, namely the use of non-random sampling methods and the lack of justification for the sample size or power calculation. The comparability domain was the least consistent, with many studies failing to control for potential confounders, e.g., parental education level, prior dental experience, and socioeconomic status. Across all studies, the outcome domain was consistently rated as adequate, supported by the use of structured, validated assessment methods including VAS used in six studies [16,20,21,22,23,26], Likert-type rating scales used in three studies [16,17,23], structured questionnaires used across all included studies, and technique ranking systems used in two studies [19,25], where parents were asked to rank behavior management techniques from most to least acceptable.

Overall, the body of evidence was rated as mostly moderate risk of bias, with three studies providing higher quality evidence and low risk of bias. These results should be considered when interpreting meta-analyses and narrative syntheses.

### 3.2. Heterogeneity

Statistical heterogeneity was assessed across all meta-analyses using the Chi^2^ test and I^2^ statistic. Heterogeneity levels varied considerably across comparisons, ranging from low to extreme, and subgroup analyses were conducted to explore potential sources of variability.

**Figure 2:**
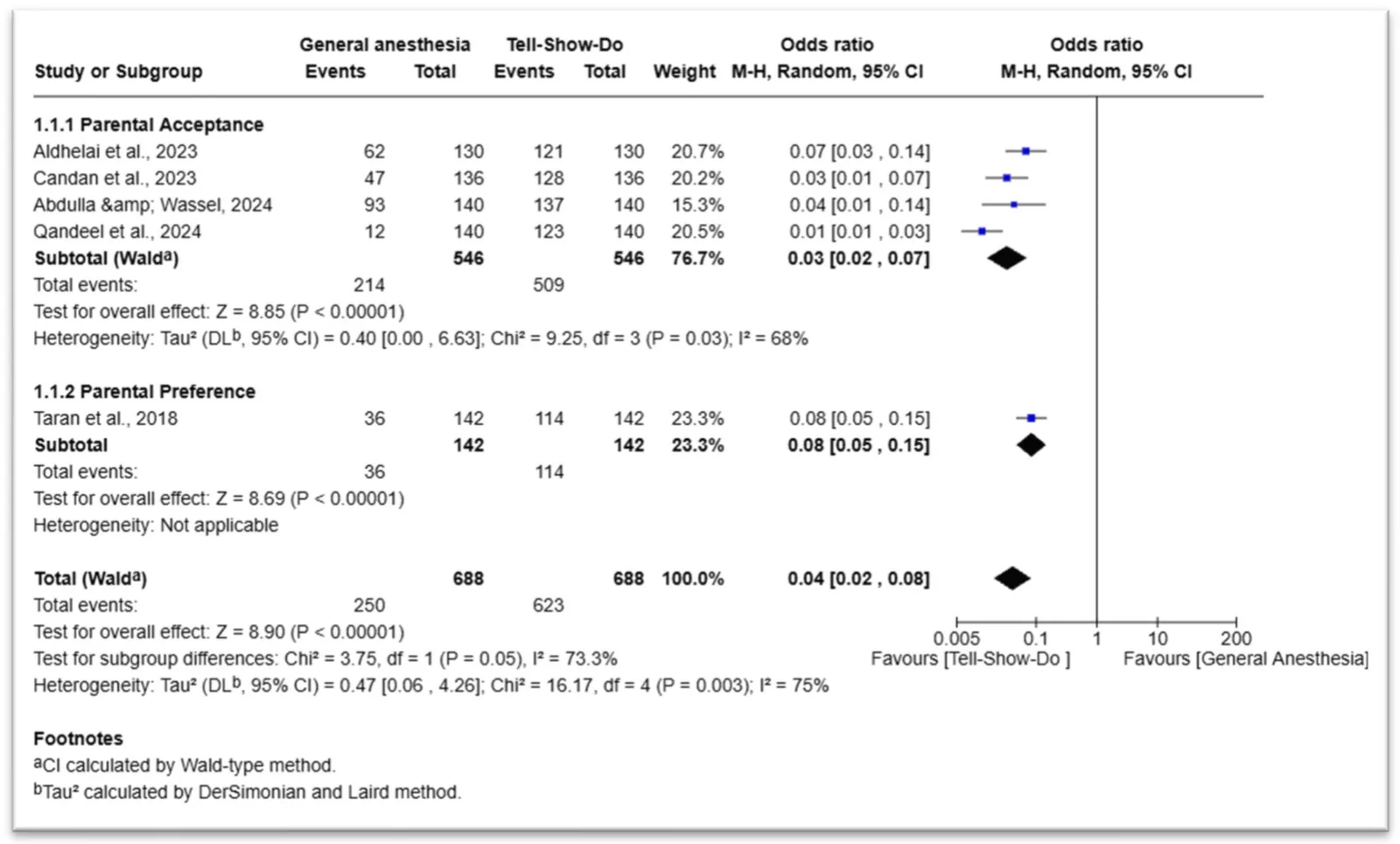
Parental acceptance and preference of general anesthesia compared to Tell-Show-Do behavior management technique (binary outcome analysis, odds ratio). Studies included: Aldhelai et al., 2023 [26]; Candan et al., 2023 [19]; Abdulla and Wassel, 2024 [16]; Qandeel et al., 2024 [23]; Taran et al., 2018 [25].

**Figure 3:**
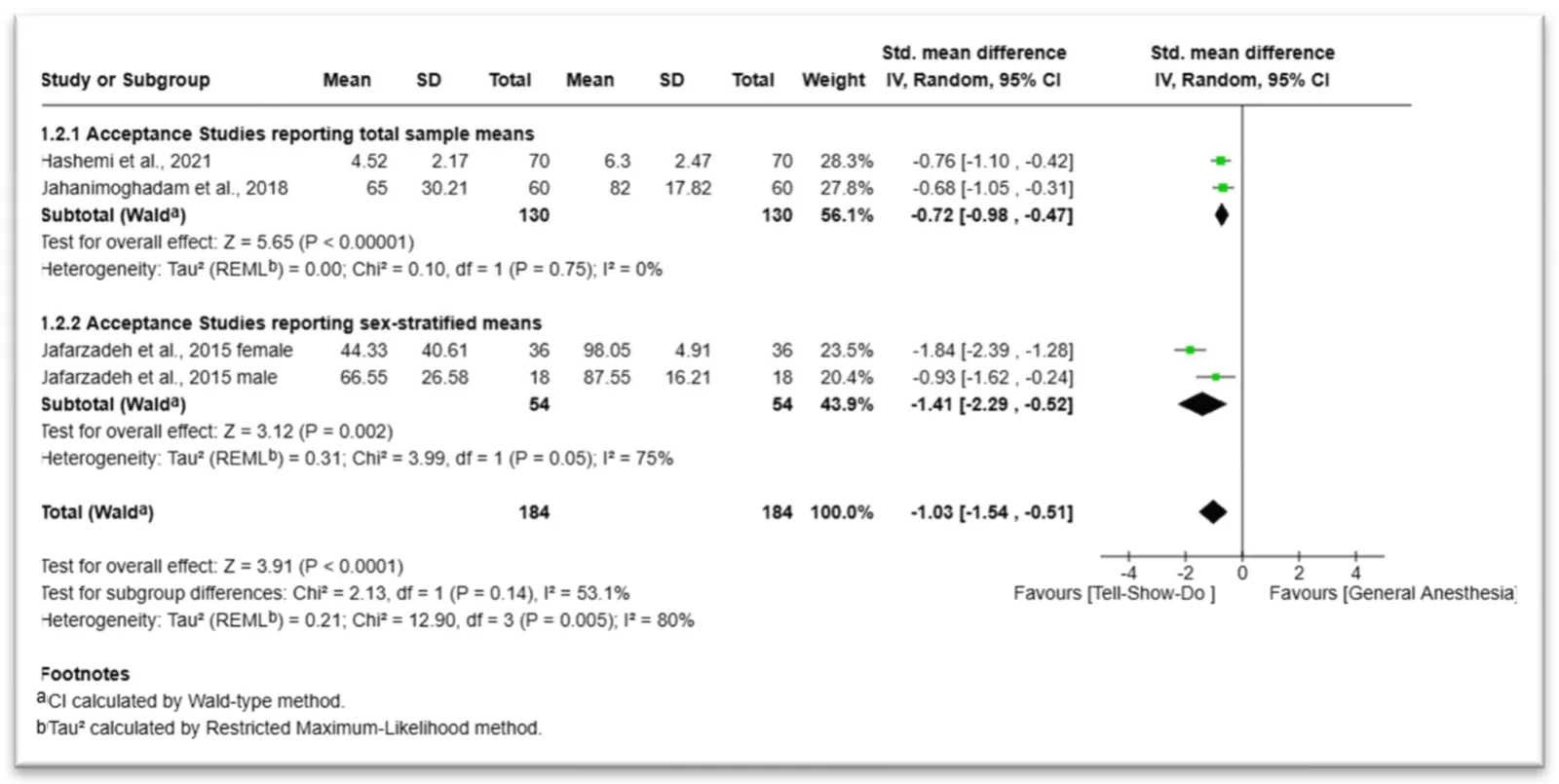
Parental acceptance of general anesthesia compared to Tell-Show-Do behavior management technique (continuous outcome analysis, standardized mean difference). Studies included: Hashemi et al., 2021 [20]; Jahanimoghadam et al., 2018 [22]; Jafarzadeh et al., 2015 [21].

For the binary analysis of GA versus TSD (Figure 2), overall heterogeneity was high (I^2^ = 75%, Chi^2^ = 16.17, df = 4, *p* = 0.003). Subgroup analysis showed that heterogeneity varied by outcome measure type: the parental acceptance subgroup showed moderate heterogeneity (I^2^ = 68%, Chi^2^ = 9.25, df = 3, *p* = 0.03), whereas the parental preference subgroup comprised a single study, so heterogeneity was not applicable. The test for subgroup differences was borderline significant (Chi^2^ = 3.75, df = 1, *p* = 0.05, I^2^ = 73.3%), suggesting that the type of outcome measure (acceptance versus preference) may partly explain the observed heterogeneity. For the continuous analysis of GA versus TSD (Figure 3), overall heterogeneity was high (I^2^ = 80%, Chi^2^ = 12.90, df = 3, *p* = 0.005). Subgroup analysis showed that studies reporting total sample means showed no heterogeneity (I^2^ = 0%), whereas studies reporting sex-stratified means showed higher heterogeneity (I^2^ = 75%), suggesting that sex-based differences in parental acceptance of GA may contribute to between-study variability. The test for subgroup differences was not statistically significant (Chi^2^ = 2.13, df = 1, *p* = 0.14, I^2^ = 53.1%).

**Figure 4:**
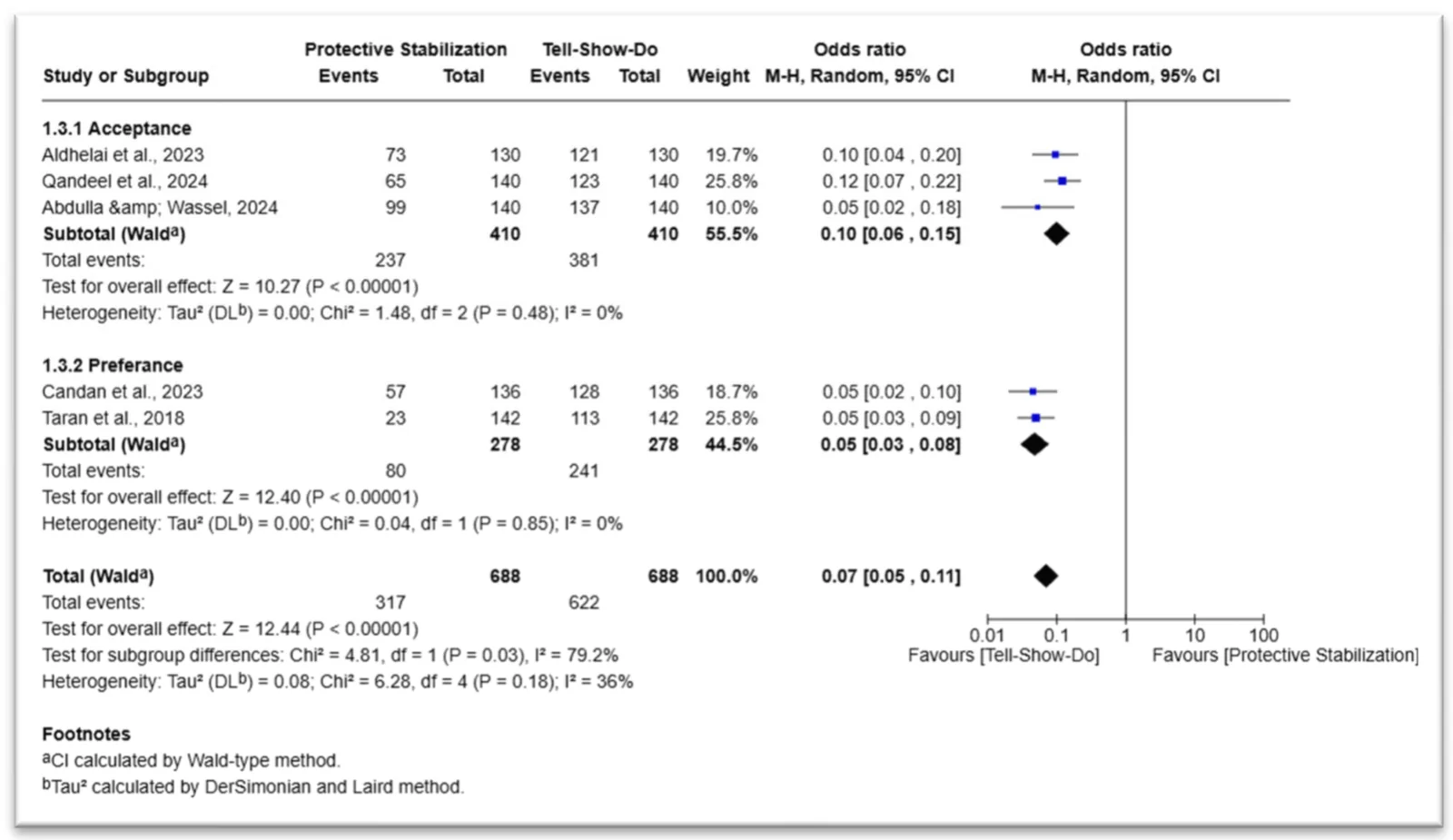
Parental acceptance and preference of protective stabilization compared to Tell-Show-Do behavior management technique (binary outcome analysis, odds ratio). Studies included: Aldhelai et al., 2023 [26]; Qandeel et al., 2024 [23]; Abdulla and Wassel, 2024 [16]; Candan et al., 2023 [19]; Taran et al., 2018 [25].

**Figure 5:**
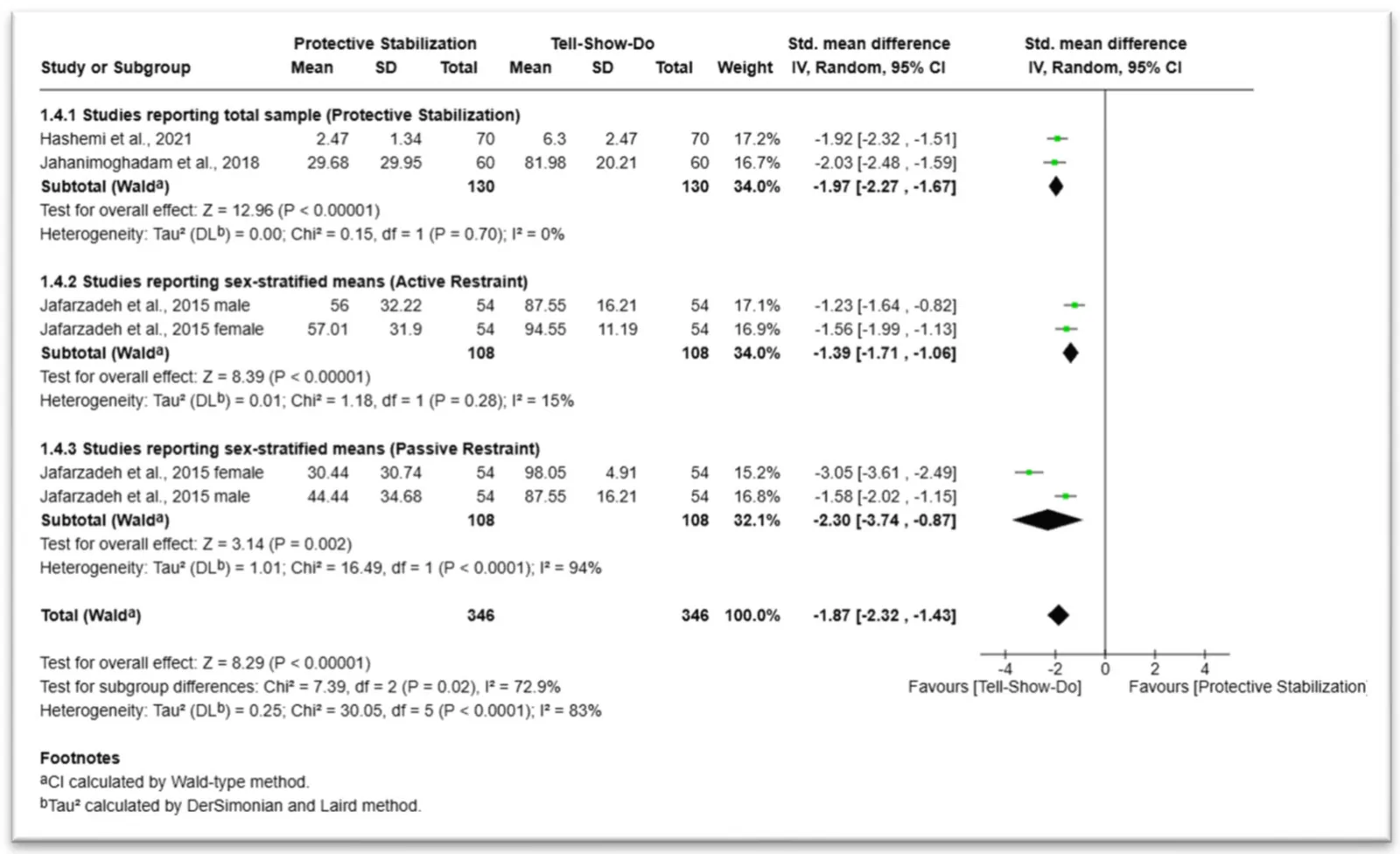
Parental acceptance of protective stabilization compared to Tell-Show-Do behavior management technique (continuous outcome analysis, standardized mean difference). Studies included: Hashemi et al., 2021 [20]; Jahanimoghadam et al., 2018 [22]; Jafarzadeh et al., 2015 [21].

For the binary analysis of protective stabilization versus TSD (Figure 4), overall heterogeneity was low to moderate (I^2^ = 36%, Chi^2^ = 6.28, df = 4, *p* = 0.18). Subgroup analysis revealed excellent consistency within each subgroup: the parental acceptance subgroup showed no heterogeneity (I^2^ = 0%, Chi^2^ = 1.48, df = 2, *p* = 0.48), and the parental preference subgroup also showed no heterogeneity (I^2^ = 0%, Chi^2^ = 0.04, df = 1, *p* = 0.85). The statistically significant test for subgroup differences (Chi^2^ = 4.81, df = 1, *p* = 0.03, I^2^ = 79.2%) indicates that the magnitude of the effect differed between acceptance and preference studies, with preference studies showing a somewhat stronger effect in favor of TSD, possibly reflecting the influence of study presentation format or cultural context on how strongly parents express a directional preference when asked to choose rather than rate. For the continuous analysis of protective stabilization versus TSD (Figure 5), overall heterogeneity was high (I^2^ = 83%, Chi^2^ = 30.05, df = 5, *p* < 0.0001). Subgroup analysis revealed marked differences across the three forms of protective stabilization examined: studies comparing total sample means for protective stabilization showed no heterogeneity (I^2^ = 0%), studies examining active restraint with sex-stratified means showed low heterogeneity (I^2^ = 15%), and studies examining passive restraint with sex-stratified means showed very high heterogeneity (I^2^ = 94%). Reflecting marked differences between male and female participants specifically, female participants showed a substantially larger acceptance gap between passive restraint and TSD than male participants [21], suggesting genuine sex-based differences in parental attitudes toward passive physical restraint. The test for subgroup differences was statistically significant (Chi^2^ = 7.39, df = 2, *p* = 0.02, I^2^ = 72.9%).

**Figure 6:**
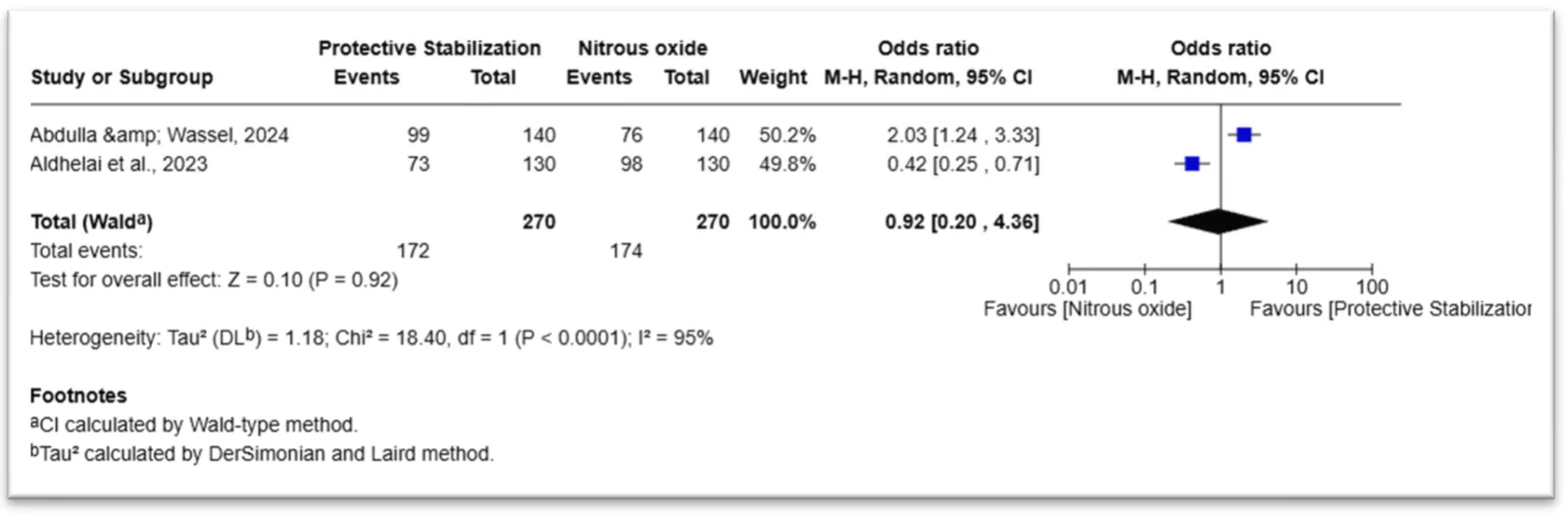
Parental acceptance of protective stabilization compared to nitrous oxide behavior management technique (binary outcome analysis, odds ratio). Studies included: Abdulla and Wassel, 2024 [16] and Aldhelai et al., 2023 [26].

**Figure 7:**
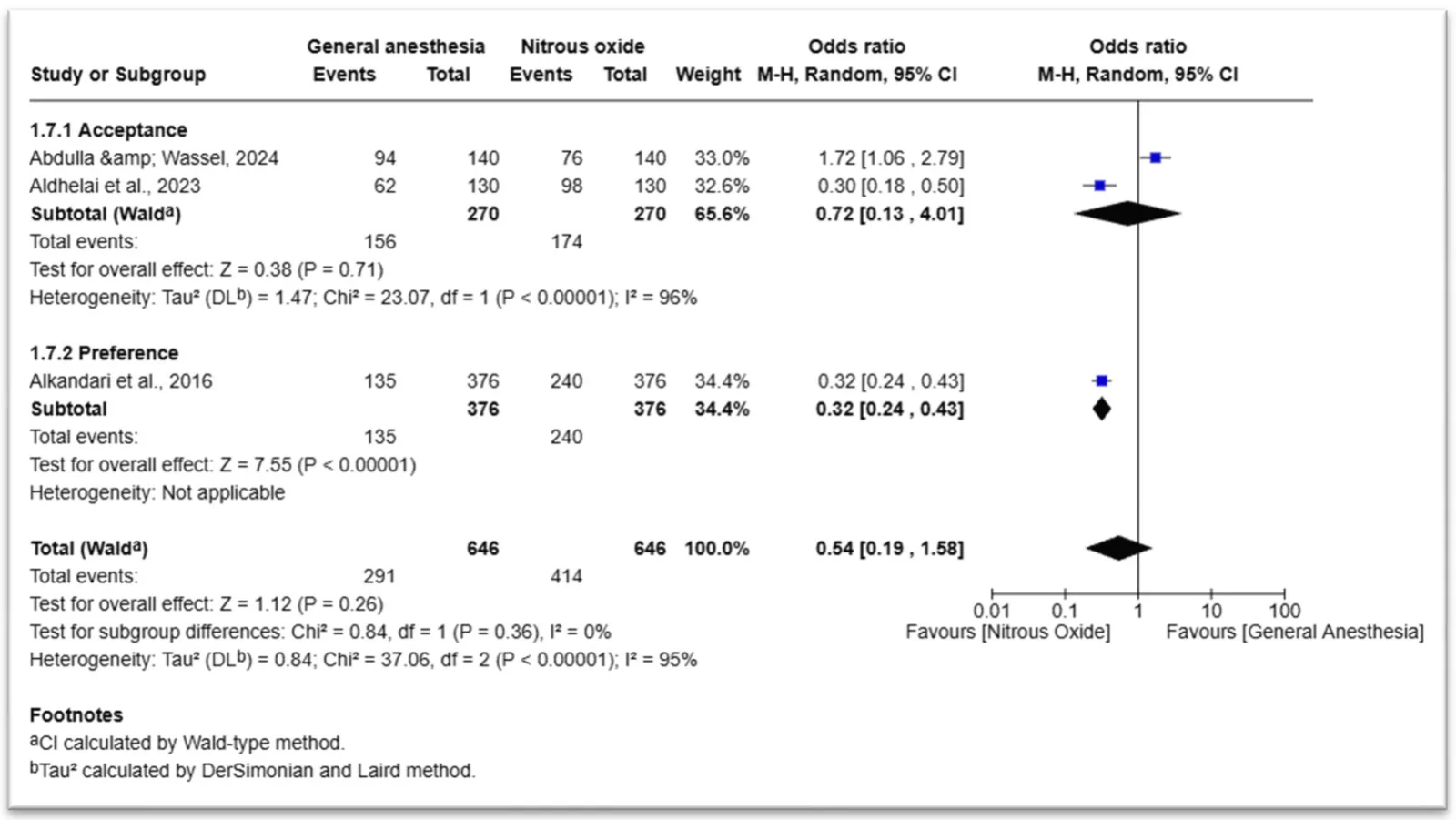
Parental acceptance and preference of general anesthesia compared to nitrous oxide behavior management technique (binary outcome analysis, odds ratio). Studies included: Abdulla and Wassel, 2024 [16]; Aldhelai et al., 2023 [26]; Alkandari et al., 2016 [18].

For the protective stabilization versus N_2_O comparison (Figure 6) and the GA versus N_2_O comparison (Figure 7), extreme heterogeneity was observed (I^2^ = 95% for both). For GA versus N_2_O, subgroup analysis revealed that the acceptance subgroup showed extreme heterogeneity (I^2^ = 96%), with two studies pointing in opposite directions: Abdulla and Wassel 2024 [16] favoring GA and Aldhelai et al. 2023 [26] favoring N_2_O, while the preference subgroup comprised a single study, Alkandari et al. 2016 [18], showing a clear preference for N_2_O over GA. The test for subgroup differences was not significant (Chi^2^ = 0.84, df = 1, *p* = 0.36, I^2^ = 0%). For protective stabilization versus N_2_O, the two contributing studies pointed in opposite directions: Abdulla and Wassel 2024 [16] favored protective stabilization, whereas Aldhelai et al. 2023 [26] favored N_2_O (I^2^ = 95%). Given the extreme heterogeneity and contradictory direction of individual study findings in both N_2_O comparisons, the pooled estimates are of very limited interpretive value, and these analyses are presented for transparency only. The contradictory findings between Abdulla and Wassel 2024 [16] and Aldhelai et al. 2023 [26] in both N_2_O comparisons are most plausibly explained by genuine contextual differences between the Bahraini and Saudi Arabian study populations, including differences in parental familiarity with N_2_O, healthcare system characteristics, and the video-based presentation formats used in each study.

Regarding sensitivity analyses, no formal sensitivity analyses such as leave-one-out analyses or restriction to low-risk-of-bias studies were conducted, as the small number of studies per comparison precluded meaningful assessment of the influence of individual studies on pooled estimates. This represents a limitation of the current review, as the robustness of the pooled estimates to the influence of individual studies cannot be formally confirmed. However, the complete absence of within-subgroup heterogeneity in the acceptance (I^2^ = 0%) and preference (I^2^ = 0%) subgroups for the protective stabilization versus TSD comparison and the consistent direction of all individual study estimates across both TSD comparisons provide indirect evidence of the robustness of the primary findings.

### 3.3. Meta-Analysis

Six meta-analyses of observational studies were conducted across four clinical comparisons to evaluate parental acceptance and preference for advanced behavior management techniques relative to basic behavior management techniques in pediatric dentistry settings across Middle Eastern populations. For comparisons with sufficient data, both binary outcome analyses, reporting results as odds ratios (OR), and continuous outcome analyses, reporting results as standardized mean differences (SMD) or mean differences (MD), were conducted using VAS score data. In all analyses, the advanced behavior management technique was designated as the intervention group, and the basic technique, either TSD or N_2_O, was designated as the reference comparator. Under this coding, an OR or mean difference below the line of no effect indicates that the advanced technique was less accepted by parents than the basic reference technique. Where applicable, studies were grouped into subgroups based on the outcome measure used, parental acceptance versus parental preference, or the data reporting format, total sample means versus sex-stratified means. The four clinical comparisons examined were GA versus TSD; protective stabilization versus TSD; GA versus N_2_O; and protective stabilization versus N_2_O.

The first meta-analysis compared parental acceptance and preference for GA versus TSD, incorporating five studies involving 688 parents in each group (Figure 2). In this analysis, GA was designated the intervention group, and TSD was the reference comparator. Studies were grouped into two subgroups based on the outcome measure used. Subgroup 1.1.1 comprised four studies that directly measured parental acceptance [16,19,23,26], while subgroup 1.1.2 comprised one study that measured parental preference [25]. In subgroup 1.1.1 (parental acceptance), the pooled odds ratio was 0.03 (95% CI: 0.02–0.07; Z = 8.85, *p* < 0.00001), indicating that the odds of parental acceptance of GA were 0.03 times the odds of accepting TSD, meaning parents were approximately 33 times more likely to accept TSD than GA. Moderate to high heterogeneity was observed (Tau^2^ = 0.40; Chi^2^ = 9.25; df = 3; *p* = 0.03; I^2^ = 68%), and a random-effects model was appropriately applied. In subgroup 1.1.2 (parental preference), Taran et al. 2018 [25] reported an odds ratio of 0.08 (95% CI: 0.05–0.15; Z = 8.69, *p* < 0.00001), indicating a strong preference for TSD over GA in the Turkish population studied. The overall pooled odds ratio across both subgroups was 0.04 (95% CI: 0.02–0.08; Z = 8.90, *p* < 0.00001), with overall high heterogeneity (Tau^2^ = 0.47; Chi^2^ = 16.17; df = 4; *p* = 0.003; I^2^ = 75%). A test for subgroup differences yielded a borderline significant result (Chi^2^ = 3.75, df = 1, *p* = 0.05, I^2^ = 73.3%), suggesting that the difference in outcome measurement acceptance versus preference may contribute to the observed heterogeneity. A random-effects model was applied throughout as a conservative approach. As shown in the forest plot, all five contributing studies consistently favored TSD over GA, with individual odds ratios ranging from 0.01 (Qandeel et al., 2024 [23]) to 0.08 (Taran et al. 2018 [25]), all pointing in the same direction. These findings provide strong and consistent evidence that TSD is substantially more accepted and preferred by parents than GA across Middle Eastern populations (Figure 2).

A complementary continuous-outcome analysis was conducted for the GA versus TSD comparison using standardized mean difference (SMD), incorporating data from four study arms across three studies reporting mean VAS scores (Figure 3). Studies were grouped into two subgroups: subgroup 1.2.1 comprised two studies reporting total sample means [20,22], and subgroup 1.2.2 comprised sex-stratified data from Jafarzadeh et al. 2015 [21], reported separately for female and male participants. In subgroup 1.2.1 (total sample means), the pooled SMD was −0.72 (95% CI: −0.98 to −0.47; Z = 5.65, *p* < 0.00001), indicating that GA acceptance scores were on average 0.72 standard deviations lower than TSD acceptance scores. Notably, this subgroup showed excellent consistency across studies (Tau^2^ = 0.00; Chi^2^ = 0.10; df = 1; *p* = 0.75; I^2^ = 0%), providing a highly reliable estimate. In subgroup 1.2.2 (sex-stratified means), the pooled SMD was −1.41 (95% CI: −2.29 to −0.52; Z = 3.12, *p* = 0.002), with higher heterogeneity (Tau^2^ = 0.31; Chi^2^ = 3.99; df = 1; *p* = 0.05; I^2^ = 75%), likely reflecting true sex-based differences in the magnitude of the acceptance gap between GA and TSD. The overall pooled SMD was −1.03 (95% CI: −1.54 to −0.51; Z = 3.91, *p* < 0.0001), indicating that across all four study arms, GA acceptance scores were on average more than one standard deviation lower than TSD acceptance scores, a large and clinically meaningful effect. Overall heterogeneity was high (Tau^2^ = 0.21; Chi^2^ = 12.90; df = 3; *p* = 0.005; I^2^ = 80%), partly attributable to differences in data reporting formats between subgroups (Chi^2^ = 2.13, df = 1, *p* = 0.14; I^2^ = 53.1%). All study arms consistently favored TSD, with no study arm showing higher acceptance for GA. Taken together with the binary analysis (Figure 2), these findings consistently and robustly confirm that parental acceptance of GA is substantially and significantly lower than acceptance of TSD across Middle Eastern populations (Figure 3).

The second meta-analysis compared parental acceptance and preference for protective stabilization versus TSD, incorporating five studies involving 688 parents in each group (Figure 4). Protective stabilization was designated as the intervention and TSD as the reference comparator. Studies were grouped into two subgroups: subgroup 1.3.1 comprised three studies measuring parental acceptance [16,23,26], and subgroup 1.3.2 comprised two studies measuring parental preference [19,25]. In subgroup 1.3.1 (parental acceptance), the pooled OR was 0.10 (95% CI: 0.06–0.15; Z = 10.27, *p* < 0.00001), indicating that parents were approximately 10 times more likely to accept TSD than PS. This subgroup showed excellent consistency with no heterogeneity (Tau^2^ = 0.00; Chi^2^ = 1.48; df = 2; *p* = 0.48; I^2^ = 0%). In subgroup 1.3.2 (parental preference), the pooled OR was 0.05 (95% CI: 0.03–0.08; Z = 12.40, *p* < 0.00001), also showing no heterogeneity (Tau^2^ = 0.00; Chi^2^ = 0.04; df = 1; *p* = 0.85; I^2^ = 0%). The overall pooled OR was 0.07 (95% CI: 0.05–0.11; Z = 12.44, *p* < 0.00001), indicating that the odds of parental acceptance of PS were 0.07 times the odds of accepting TSD, meaning parents were approximately 14 times more likely to accept TSD than PS. A statistically significant subgroup difference was observed (Chi^2^ = 4.81, df = 1, *p* = 0.03; I^2^ = 79.2%), suggesting that the magnitude of effect differed between acceptance and preference studies, with preference studies showing a somewhat stronger effect in favor of TSD. Overall heterogeneity was low (Tau^2^ = 0.08; Chi^2^ = 6.28; df = 4; *p* = 0.18; I^2^ = 36%), and a random-effects model was applied. All five studies consistently favored TSD over PS, providing strong and consistent evidence across Middle Eastern populations (Figure 4).

A complementary continuous outcome analysis was conducted for the PS versus TSD comparison using standardized mean difference (SMD), incorporating data from three studies across six study arms (Figure 5). Studies were organized into three subgroups reflecting different forms of protective stabilization: subgroup 1.4.1 comprised two studies reporting total sample means for protective stabilization [20,22]; subgroup 1.4.2 comprised sex-stratified data from Jafarzadeh et al. 2015 for active restraint [20]; and subgroup 1.4.3 comprised sex-stratified data from Jafarzadeh et al. 2015 for passive restraint [21]. In the subgroup 1.4.1 protective stabilization total sample, the pooled SMD was −1.97 (95% CI: −2.27 to −1.67; Z = 12.96, *p* < 0.00001), with no heterogeneity (Tau^2^ = 0.00; Chi^2^ = 0.15; df = 1; *p* = 0.70; I^2^ = 0%), indicating a large and highly consistent effect favoring TSD. In subgroup 1.4.2, active restraint sex-stratified, the pooled SMD was −1.39 (95% CI: −1.71 to −1.06; Z = 8.39, *p* < 0.00001), with low heterogeneity (I^2^ = 15%). In subgroup 1.4.3 (passive restraint—sex-stratified), the pooled SMD was −2.30 (95% CI: −3.74 to −0.87; Z = 3.14, *p* = 0.002), with very high heterogeneity (Tau^2^ = 1.01; Chi^2^ = 16.49; df = 1; *p* < 0.0001; I^2^ = 94%), reflecting marked differences between male and female participants in the magnitude of acceptance gap for passive restraint versus TSD. The overall pooled SMD was −1.87 (95% CI: −2.32 to −1.43; Z = 8.29, *p* < 0.00001), indicating that across all forms of protective stabilization, acceptance scores were on average 1.87 standard deviations lower than TSD acceptance scores—a very large effect. A significant subgroup difference was observed (Chi^2^ = 7.39, df = 2, *p* = 0.02; I^2^ = 72.9%), reflecting differing effect magnitudes across the three forms of PS. Overall heterogeneity was high (Tau^2^ = 0.25; Chi^2^ = 30.05; df = 5; *p* < 0.0001; I^2^ = 83%), attributable to heterogeneity within the passive restraint subgroup. Consistent with the binary analysis (Figure 4), all study arms showed lower acceptance scores for PS compared with TSD, reinforcing the conclusion that TSD is substantially more accepted by parents than any form of protective stabilization (Figure 5).

The comparison between GA and N_2_O incorporated three studies involving 646 parents in each group (Figure 7). GA was designated as the intervention and N_2_O as the reference comparator. Studies were grouped into two subgroups: subgroup 1.7.1 comprised two studies measuring parental acceptance [16,26], and subgroup 1.7.2 comprised one study measuring parental preference [18]. In subgroup 1.7.1, parental acceptance, the pooled OR was 0.72 (95% CI: 0.13–4.01; Z = 0.38, *p* = 0.71), which was not statistically significant. Critically, this subgroup demonstrated extreme heterogeneity (Tau^2^ = 1.47; Chi^2^ = 23.07; df = 1; *p* < 0.00001; I^2^ = 96%), with the two contributing studies pointing in opposite directions. Abdulla & Wassel 2024 [16] favored GA (OR = 1.72), while Aldhelai et al. 2023 [26] favored N_2_O (OR = 0.30), rendering the pooled estimate uninterpretable as a reliable summary effect. In subgroup 1.7.2 (parental preference), Alkandari et al. 2016 [18] reported an OR of 0.32 (95% CI: 0.24–0.43; Z = 7.55, *p* < 0.00001), clearly favoring N_2_O over GA. The overall pooled OR was 0.54 (95% CI: 0.19–1.58; Z = 1.12, *p* = 0.26), which was not statistically significant, with extreme overall heterogeneity (Tau^2^ = 0.84; Chi^2^ = 37.06; df = 2; *p* < 0.00001; I^2^ = 95%). No significant subgroup difference was detected (Chi^2^ = 0.84, df = 1, *p* = 0.36; I^2^ = 0%).

The comparison between PS and N_2_O incorporated two studies involving 270 parents in each group (Figure 6). PS was designated as the intervention and N_2_O as the reference comparator. Abdulla & Wassel 2024 [16] reported an OR of 2.03 (95% CI: 1.24–3.33), favoring PS over N_2_O. In contrast, Aldhelai et al. 2023 [26] reported an OR of 0.42 (95% CI: 0.25–0.71), favoring N_2_O over PS. These two studies pointed in completely opposite directions. The pooled OR was 0.92 (95% CI: 0.20–4.36; Z = 0.10, *p* = 0.92), which was not statistically significant, with extreme heterogeneity (Tau^2^ = 1.18; Chi^2^ = 18.40; df = 1; *p* < 0.0001; I^2^ = 95%). At I^2^ = 95%, the between-study variance completely dominates the analysis, and the pooled OR is essentially uninterpretable. This analysis is presented for transparency only. The divergent findings between the two contributing studies, one Bahraini and one Saudi Arabian, likely reflect genuine contextual and cultural differences in parental familiarity with and attitudes toward N_2_O and protective stabilization in these two distinct healthcare settings. No meaningful conclusion about the relative parental acceptance of PS versus N_2_O can be drawn from the available evidence (Figure 6).

## 4. Discussion

This systematic review and meta-analysis of observational studies evaluated parental acceptance of various BGTs in pediatric dentistry across Middle Eastern populations. While the available evidence suggests a general tendency toward greater parental acceptance of non-invasive approaches, particularly TSD, over more advanced or restrictive methods such as GA, sedation, and physical or protective restraint, these findings should be interpreted cautiously in light of the methodological limitations of the included studies, the predominantly moderate risk of bias, and the substantial heterogeneity observed across several analyses. The findings are therefore considered preliminary and hypothesis-generating rather than definitive and should not be interpreted as a basis for formal evidence-based clinical recommendations.

TSD was the most consistently accepted technique among parents. Across the included studies, acceptance rates for TSD ranged from 88% to 98% [16,21,22,26]. Compared with GA, physical restraint, or sedation, TSD was repeatedly ranked highest by parents, indicating widespread favorability for behavioral techniques that emphasize verbal guidance, emotional comfort, and trust. The meta-analyses demonstrated consistent and significant findings across both acceptance and preference outcome measures. For GA versus TSD, parental acceptance studies showed a pooled OR of 0.03 (95% CI: 0.02–0.07), indicating parents were approximately 33 times more likely to accept TSD than GA, and parental preference studies similarly favored TSD (OR = 0.08, 95% CI: 0.05–0.15), with an overall pooled OR of 0.04 (95% CI: 0.02–0.08). For protective stabilization versus TSD, parental acceptance studies showed a pooled OR of 0.10 (95% CI: 0.06–0.15), indicating that parents were approximately 10 times more likely to accept TSD than protective stabilization, with parental preference studies also favoring TSD (OR = 0.05, 95% CI: 0.03–0.08) and an overall pooled OR of 0.07 (95% CI: 0.05–0.11). Both comparisons were further supported by large standardized effect sizes in the continuous-outcome analyses, favoring TSD for GA (SMD = −1.03, 95% CI: −1.54 to −0.51) and protective stabilization (SMD = −1.87, 95% CI: −2.32 to −1.43). These results are consistent with the AAPD guidelines, which recommend TSD and other communicative strategies as first-line techniques in the management of pediatric patients [27].

Acceptance of N_2_O varied more widely. While several studies [18,19,26] reported that N_2_O was more accepted than GA or physical restraint, others [15] found lower acceptance. The meta-analyses comparing GA versus N_2_O and PS versus N_2_O both demonstrated extreme heterogeneity (I^2^ = 95% for both comparisons), rendering the pooled estimates of very limited interpretive value. Rather than relying on pooled odds ratios, individual study findings provide a more informative picture of N_2_O acceptance patterns across the included populations.

Some studies reported higher N_2_O acceptance relative to GA and physical restraint. Alkandari et al. 2016 [18] reported N_2_O acceptance of 64% compared to 36% for GA, and Aldhelai et al. 2023 [26] reported N_2_O acceptance of 75.4% compared to 56.2% for protective stabilization and 47.7% for GA. In contrast, Abdulla & Wassel 2024 [16] notably reported that N_2_O was less accepted than both physical restraint (54% vs. 71%, *p* = 0.003) and GA (54% vs. 67%, *p* = 0.02), representing the only study where N_2_O was less preferred than other advanced techniques, a finding that likely reflects specific cultural and clinical characteristics of the Bahraini context.

Of particular interest is the comparison reported by Sabbagh et al. (2021) [24], conducted in Saudi Arabia among parents with firsthand experience of the evaluated techniques. In that study, parental acceptance of the papoose board was 100%, compared with 96.2% for N_2_O, with no statistically significant difference between the two techniques (*p* = 1.00). This notably high acceptance of the papoose board, a technique commonly regarded as invasive and restrictive, may be explained by the study’s treatment-based context, in which parents had prior direct experience with the technique and could evaluate it based on actual outcomes rather than video or image presentations. However, the combined use of the papoose board with N_2_O resulted in a significantly lower acceptance rate (81.2%) compared with N_2_O alone (*p* = 0.04), suggesting that combining techniques may reduce parental acceptance even when individual techniques are well accepted in isolation. Similarly, Bagher et al. 2023 [28], also conducted in Saudi Arabia, examined parental preference among 306 parents whose children had previously undergone comprehensive dental treatment under GA. In that study, 55.6% of parents preferred to repeat GA for future dental treatment. This notably higher GA preference rate, compared with the 9% to 47.7% acceptance rates reported in studies where parents had no prior GA experience, strongly suggests that direct personal experience with GA may substantially increase parental acceptance of this advanced technique. Together, these two experience-based studies highlight an important moderating role of prior parental experience on acceptance of advanced behavior management techniques. This finding has significant clinical implications, suggesting that parental education and discussion of actual treatment experiences may help bridge the gap between initial reluctance toward advanced techniques and post-treatment acceptance.

These divergent findings confirm that parental acceptance of N_2_O is highly context-dependent and cannot be meaningfully summarized by a single pooled estimate. The variability likely reflects differences in study presentation formats (video-based vs. questionnaire-based) and cultural attitudes toward pharmacological interventions. Similar context-dependent patterns have been reported globally, in which acceptance of N_2_O is closely linked to parental understanding and prior exposure [1].

Cultural factors influenced parental perceptions. Studies from Iran, Turkey, and Egypt reported high acceptance of TSD and more reserved attitudes toward pharmacological interventions [20,23,25]. In contrast, in Saudi Arabia and Kuwait, where sedation and GA may be more familiar, parents displayed a slightly broader range of acceptance [18,24]. These regional diversities strengthen the importance of culturally sensitive communication and patient-centered education when introducing behavior management options. There is a variation in parental acceptance across different countries, considering the potential influence of cultural and religious beliefs, differences in healthcare access, and the financial implications associated with sedation and GA. This strategy is highly recommended and supported by international guidelines. The AAPD agreed that behavior guidance should be adjusted to the child’s developmental level, medical background, family expectations, and parental thoughts, with joint decision-making forming a core component of ethical pediatric dental care [27]. Additionally, the World Health Organization (WHO) encourages family-centered and culturally responsive healthcare, emphasizing the crucial role of appropriate interactive communication, parental involvement in decision-making, and culturally adopted care strategies tailored to local cultural contexts [29].

In the United States, parental acceptance of pharmacological behavior management techniques increased over time, with sedation and the TSD technique being the most widely accepted, whereas passive restraint (papoose board) and the hand-over-mouth technique were the least accepted [30]. In Spain, the TSD technique was the most widely accepted behavior management approach, followed by active restraint, N_2_O sedation, and GA, whereas the hand-over-mouth technique and the papoose board were the least accepted. In contrast, studies from Germany indicate that N_2_O sedation is generally the most preferred advanced behavior management technique [25].

This review does have limitations. Most included studies were cross-sectional, limiting the ability to infer causality. There was also considerable heterogeneity across studies, particularly those involving N_2_O, likely due to differences in parental backgrounds, presentation formats, and the measurement tools (e.g., Likert vs. VAS scales). Publication bias was not assessed using funnel plots because the number of included studies was limited. For the continuous outcome analyses, two studies (Jahanimoghadam et al. 2018 [22] and Jafarzadeh et al. 2015 [21]) used different VAS scales across comparisons, making direct pooling of mean differences inappropriate and contributing to the high heterogeneity observed in the continuous GA versus TSD analysis. Standardized mean differences were used where applicable to partially address scale incompatibility. No formal sensitivity analyses were conducted due to the limited number of studies per comparison, which prevents formal confirmation of the robustness of the pooled estimates to the influence of individual studies. Additionally, although the regional focus of this review enhances cultural specificity, it may restrict generalizability to other global populations.

Despite these limitations, the available evidence suggests that parents in Middle Eastern populations tend to favor behavioral interventions, such as TSD, over more invasive or restrictive approaches. These preliminary findings may inform clinical awareness and encourage pediatric dentists to consider parental perspectives when discussing behavior management options, while recognizing that individual clinical decisions must account for the child’s specific needs, the clinical context, and the practitioner’s professional judgment. Formal clinical recommendations await higher-quality longitudinal and multicenter evidence.

### Implications for Future Research

Future studies should examine longitudinal parental perceptions rather than cross-sectional observations. Monitoring changes in parental attitudes over time, particularly after firsthand experiences with various BGTs, would improve the understanding of acceptance patterns and satisfaction rates. Future research should also prioritize standardized outcome measurement tools to enable more reliable quantitative synthesis, broader geographic representation within and beyond the Middle East, and exploration of factors such as parental education level, cultural background, prior dental experience, and child temperament that may influence acceptance patterns. High-quality randomized or prospective studies comparing parental acceptance before and after experiencing specific BGTs would substantially strengthen the evidence base.

## 5. Conclusions

This systematic review and meta-analysis synthesized evidence from 11 cross-sectional observational studies conducted across Middle Eastern populations, demonstrating consistently lower parental acceptance of advanced behavior management techniques compared with basic techniques. Parental acceptance of GA was approximately 33 times lower than TSD (OR = 0.03, 95% CI: 0.02–0.07) and protective stabilization approximately 10 times lower (OR = 0.10, 95% CI: 0.06–0.15), with both findings further supported by large continuous effect sizes (GA vs. TSD: SMD = −1.03; protective stabilization vs. TSD: SMD = −1.87). Acceptance of advanced techniques relative to N_2_O was highly variable and context-dependent, with extreme heterogeneity (I^2^ = 95%) precluding reliable conclusions.

This systematic review shows that the high parental acceptance of the TSD technique and the correspondingly low acceptance of advanced techniques such as GA and protective stabilization is primarily derived from cross-sectional studies and should be interpreted in light of the moderate risk of bias and substantial heterogeneity among the included studies. These findings are therefore considered preliminary and may inform clinical awareness among pediatric dental practitioners rather than serve as a basis for formal clinical recommendations.

There is a need for further high-quality, longitudinal, and multicenter studies to strengthen the evidence base for culturally informed behavior guidance in pediatric dental practice across the Middle East.

## Figures and Tables

**Table 1:** Comparison of parental acceptance/preference between advanced behavior guidance techniques and Tell-Show-Do (TSD).

Author Year, Country	Study Design	Simple Size (Male/Female)	Children Age (years)	Method of Assessment	Method Tools	Outcome		
					Exposure	Exposure N (%)	Control N (%)	*p* Value
Jafarzadeh et al., 2015 Iran [21]	Cross-sectional Structured study Video-based	54 parents (18/36)	Not mentioned	Parental acceptance: interview questionnaire VAS	Active Restraint	Male: 56 ± 32.27 Female: 57.52 ± 32.22	Male: 87.55 ± 16.21 Female: 98.05 ± 4.91	<0.001
					Oral Sedation	Male: 65.66 ± 36 Female: 49.11 ± 36.35		<0.001
					GA	Male: 66.55 ± 26.58 Female: 44.33 ± 40.61		<0.001
					Passive restraint	Male: 44.44 ± 34.68 Female: 30.44 ± 30.74		<0.001
Jahanimoghadam et al., 2018 Iran [22]	Cross -sectional descriptive-analytic study, Video-based	60 parents (19/41)	3–15	Parental acceptance questionnaire, VAS	G.A	Mean: 65 ± 30.21^$^	Mean: 82 ± 17.82^$^	0.001
					Physical Restraint	Mean: 29.44 ± 33.31^$^		0.001
Taran et al., 2018 Turkey [25]	Cross-sectional study Video-based	142 parents (68/74)	3–12	Parental preference questionnaire	Sedation	48/142 (33.8)	114/142 (80.3)	<0.001
					G.A	36/142 (25.4)		<0.001
					Protective Stabilization	24/142 (16.9)		<0.001
Hashemi et al., 2021 Iran [20]	Cross-sectional descriptive-analytical images-based	70 parents (18/52)	<12	Parental acceptance questionnaire, VAS scores	Sedation	Mean: 3.92 ± 1.13	Mean: 6.3 ± 2.47	<0.001
					G.A	Mean: 4.52 ± 2.17		<0.001
					Physical Restrain	Mean: 2.47 ± 1.34		<0.001
Aldhelai et al., 2023 Saudi Arabia [26]	Cross-sectional Video-based	130 parents (86/44)	3–12	Parental acceptance questionnaire, VAS	G.A	62/130 (47.7)	121/130 (93.1)	<0.001
					Protective Stabilization	73/130 (56.2)		<0.001
Candan et al., 2023 Turkey [19]	Cross-sectional Video-based	136 parents (37/99)	Mean: 7.88 ± 2.70	Parental preference, self-administered questionnaire	N_2_O & sedation	68/136 (50)	128/136 (94.1)	<0.001
					G.A	41/136 (30.1)		<0.001
					Protective Stabilization	57/136 (41.9)		<0.001
Abdulla & Wassel, 2024 Bahrain [16]	Cross-sectional Video-based	140 parents	2–12 years	Parental acceptance, face-to-face interview questionnaire, VAS, Likert scale	G.A	93/140 (67)	137/140 (98)	<0.001
					Physical Restrain	99/140 (71)		<0.001
Qandeel et al., 2024 Egypt [23]	Cross-sectional Video-based	140 parents	2–12 years	Parental acceptance, face-to-face interviews questionnaire, VAS, Likert scale	Physical Restrain	65/140 (47)	123/140 (88)	<0.001
					G.A	12/140 (9)		<0.001

VAS: visual analog scale, TSD: Tell-Show-Do, G.A: General anesthesia, N_2_O: nitrous oxide, ^$^ Binary parental acceptance was determined from the scores assigned by parents to each behavior management technique. Scores corresponding to 75% or more were classified as acceptance, whereas scores below 75% were classified as non-acceptance.

**Table 2:** Comparison of parental acceptance between advanced behavior guidance technique and nitrous oxide (N_2_O).

Author Year, Country	Study Design	Simple Size	Children Age (years)	Method of Assessment	Method Tools	Outcome		
					Exposure	Exposure N (%)	Control N (%)	*p* Value
Alkandari et al., 2016 Kuwait [18]	Cross-sectional survey	376 Parent (179M/197F) 367 children (184M/182F)	Mean: 5.9	Parental preference: Self-administered questionnaire	G.A	135/376 (36) ***	248/376 (66) ** 240/376 (64) ***	<0.001
Al Zoubi et al., 2021 Jordan [17]	Cross-sectional study, photo-based	99 parents (15M/84F)	Not mention	Parental acceptance questionnaire, Likert scale	Passive restraint	Mean: 2.52 ± 1.50	Mean: 3.22 ± 1.50	0.001
					Active restraint	Mean: 3.08 ± 1.33		0.4
					G.A	Mean: 2.11 ± 1.30		<0.001
Sabbagh et al.,2021 Saudi Arabia [24]	Cross-sectional prospective treatment-based	132 parents	2–12	Parental acceptance questionnaire after parents experienced the behavior management technique	Papoose Board	10/10 (100)	102/106 (96.2)	1
					N_2_O and Papoose	13/16 (81.2)		0.04
Aldhelai et al., 2023 Saudi Arabia [26]	Cross-sectional Video-based	130 parents (86M/44F)	3–12	Parental acceptance questionnaire, VAS, Likert scale	Protective Stabilization	73/130 (56.2)	98/130 (75.4)	0.003
					G.A	62/130 (47.7)		<0.001
Abdulla & Wassel, 2024 Bahrain [16]	Cross-sectional Video-based	140 parents	2–12	Parental acceptance, face-to-face interview questionnaire, VAS, Likert scale	Physical Restrain	99/140 (71)	76/140 (54)	0.003
					G.A	94/140 (67)		0.02

VAS: visual analog scale, G.A: general anesthesia, N_2_O: nitrous oxide, ** Parental acceptance: parents who indicated willingness to accept N_2_O sedation if recommended by the treating dentist, *** parental preference.

**Table 3:** Risk of bias summary: review authors’ judgments for each included study according to each risk of bias item.

Study	Selection	Comparability	Outcome	Overall Score	Risk Category
Jafarzadeh et al., 2015 [21]	1	2	2	5	Moderate
Alkandari et al., 2016 [18]	3	1	2	6	Moderate
Jahanimoghadam et al., 2018 [22]	2	1	2	5	Moderate
Taran et al., 2018 [25]	3	1	2	6	Moderate
Hashemi et al., 2021 [20]	2	1	2	5	Moderate
Al Zoubi et al., 2021 [17]	2	2	2	6	Moderate
Sabbagh et al., 2021 [24]	4	2	2	8	Low
Aldhelai et al., 2023 [26]	3	2	2	7	Low
Candan et al., 2023 [19]	2	2	2	6	Moderate
Abdulla & Wassel, 2024 [16]	3	1	2	6	Moderate
Qandeel et al., 2024 [23]	4	1	2	7	Low

Selection domain (maximum 4 stars): assessed representativeness of the sample, sample size justification, non-respondent description, and ascertainment of exposure. Comparability domain (maximum 2 stars): assessed control for confounders in design or analysis. Outcome domain (maximum 3 stars): assessed appropriateness of outcome assessment and completeness of outcome data. Total scores: >6 = low risk of bias; 4–6 = moderate risk; ≤3 = high risk.

## Data Availability

No new data were created or analyzed in this study.

## Supplementary Materials

The following supporting information can be downloaded at: https://www.mdpi.com/article/10.3390/healthcare14172729/s1, File S1: PRISMA 2020 Checklist.

## Abbreviations

The following abbreviations are used in this manuscript:

BGT	behavior guiding technique
AAPD	American Academy of Pediatric Dentistry
TSD	Tell-Show-Do
N_2_O	nitrous oxide
GA	general anesthesia
SHCN	special health care needs
ASA	American Society of Anesthesiologists
PRISMA	Preferred Reporting Items for Systematic Reviews and Meta-analyses
PROPERO	International Prospective Register of Systematic Reviews
NOS	Newcastle–Ottawa Scale
OR	odds ratio
VAS	visual analog scale
SMD	standardized mean differences
WHO	World Health Organization
